# Protein Lysine Lactylation in DNA Damage Response: Regulatory Mechanisms and Emerging Functions

**DOI:** 10.3390/biom16101528

**Published:** 2026-10-20

**Authors:** Xinke Pei, Jinhui Zhang, Yue Gao, Zuo Pan, Yue Wang, Luyu Wang, Zhenguo Wang, Guo-Bin Ding, Roland H. Stauber, Ting Ni, Shuailin Hao

**Affiliations:** 1 State Key Laboratory of Reproductive Regulation and Breeding of Grassland Livestock, Institutes of Biomedical Sciences, School of Life Sciences, Inner Mongolia University, Hohhot 010070, China; 22692021@mail.imu.edu.cn (X.P.);; 2 Department of Otorhinolaryngology Head and Neck Surgery, Molecular and Cellular Oncology, University Medical Center Mainz, 55131 Mainz, Germany; rstauber@uni-mainz.de

**Keywords:** lysine lactylation, DNA damage response, DNA repair, lactate metabolism, genome stability

## Abstract

Genomic DNA is constantly exposed to endogenous and exogenous stresses that can cause different types of DNA damage. DNA double-strand breaks (DSBs) are among the most damaging forms of DNA lesions, as persistent breaks can compromise genome stability. In response to such damage, cells activate DNA damage response (DDR) pathways that detect lesions and promote their repair. Lysine lactylation (Kla), a recently identified lysine acylation modification, links cellular metabolism and DNA repair. Recent evidence suggests that Kla may influence DDR by modulating the stability, interactions, and activity of DNA repair proteins. This review discusses recent advances in our understanding of lactylation in homologous recombination (HDR) and non-homologous end joining (NHEJ). We focus on MRE11, NBS1, BLM, and XLF lactylation and their roles in DNA repair and tumor chemoresistance. These findings suggest that lactylation can affect genome stability, tumor progression, and responses to cancer therapy by regulating key DDR factors. However, how lactylation is regulated during DDR and how it affects DNA repair remain largely unclear. Further studies are needed to elucidate how DNA damage-induced lactylation can be regulated, the functions of lactylation writers and erasers, and the significance of lactylation in various disease contexts.

## 1. Introduction

Genomic DNA is the primary repository of human genetic information, and its stability is crucial for normal development, reproduction, and the maintenance of essential physiological functions. However, genomic DNA is constantly exposed to environmental and intracellular sources of damage. These include ionizing and ultraviolet radiation, chemical toxins, reactive oxygen species (ROS) produced during cellular metabolism, replication stress, and transcription-associated abnormalities. These can cause diverse types of DNA damage [1,2,3,4,5,6]. Failure to repair DNA damage efficiently can lead to genomic instability and the accumulation of genetic mutations, contributing to the development of various diseases, including cancer [5,7]. To protect the genome from endogenous and exogenous damage, cells evolved a conserved DDR system [8,9]. DDR detects DNA lesions and initiates damage signaling; it controls cell cycle progression and recruits DNA repair factors to damage sites [10,11,12,13]. It coordinates DNA repair with other cellular processes, including DNA replication and transcription [10]. Efficient DNA repair and maintenance of genome stability therefore depend on proper DDR activation [3]. When DDR signaling or repair is impaired, DNA damage can accumulate, and genomic instability may increase, potentially contributing to disease development [3].

DSBs are among the most severe forms of DNA damage and strongly induce DDR [14,15]. Failure to correctly identify or repair DSBs can lead to loss or rearrangement of genetic information [3]. DSBs can also cause mutations and chromosomal abnormalities, including deletions, duplications, inversions, and translocations [16,17]. These changes can promote cancer development and progression [16].

The phosphatidylinositol 3-kinase-related kinase (PIKK) family plays a central role in DDR signaling [1,18]. It includes three major kinases: ataxia–telangiectasia mutated (ATM), Rad3-related protein (ATR), and DNA-dependent protein kinase catalytic subunit (DNA-PKcs) [13]. ATM and DNA-PKcs primarily mediate signaling in response to DSBs [19]. In contrast, ATR mainly responds to replication stress and persistent DNA damage [19]. Upon activation, these kinases phosphorylate downstream proteins and regulate cell cycle checkpoints, DNA repair, and apoptosis [20,21]. Post-translational modifications (PTMs) also contribute to DDR regulation [22]. PTMs can alter DDR signaling, repair factor recruitment, and DNA repair protein activity [22,23]. Through these effects, PTMs help cells respond to DNA damage and maintain genome stability [22].

Following DNA DSBs, cells rapidly activate the DDR to detect and repair damaged DNA. As an early sensor of DSBs, the MRE11–RAD50–NBS1 (MRN) complex recognizes DNA ends at break sites and promotes ATM recruitment and activation in response to DNA damage [24,25,26]. DSB formation also triggers phosphorylation of histone H2AX at serine 139, leading to the accumulation of γH2AX foci around damaged DNA [27,28,29,30]. These foci serve as a widely used marker of DNA damage and facilitate the recruitment of additional DDR factors [27,28,29,30]. Following DNA damage, cells engage distinct repair pathways depending on the cell cycle phase and the characteristics of the DNA lesion. HDR is an accurate pathway for repairing DSBs that mainly uses the sister chromatid as a template and is preferentially active during the S and G2 phases of the cell cycle [11,31,32,33,34,35]. Impaired HDR renders cells more sensitive to ionizing radiation, DNA-damaging chemotherapeutic agents, and poly(ADP-ribose) polymerase (PARP) inhibitors [36]. NHEJ provides a faster alternative and functions throughout the cell cycle [37]. It joins broken DNA ends directly without requiring a homologous template; however, this mechanism may result in sequence alterations [38]. Core NHEJ factors, including the Ku70–Ku80 heterodimer, XLF, and DNA ligase IV, mediate the recognition and direct re-ligation of DSB ends [35,39,40,41]. Although NHEJ deficiency compromises DSB repair and sensitizes cells to genotoxic stress, it generally does not confer the PARP inhibitor hypersensitivity characteristic of HDR deficiency [36]. The NHEJ machinery exhibits considerable functional flexibility, allowing diverse DNA end structures to be processed and repaired through multiple possible outcomes, even when the initial DNA ends are identical [42].

Increasing evidence suggests that PTMs play critical roles in regulating DNA repair [43]. The importance of PTMs in DDR regulation is reported extensively in studies on phosphorylation-dependent signaling events following DSB induction. This shows that dynamic phosphorylation and dephosphorylation of key DDR factors are essential for coordinating DNA damage signaling and repair [23,44,45]. With continued advances in this field, an increasing number of PTMs have been identified beyond phosphorylation as important regulators of DSB-induced damage responses, including ubiquitination, ubiquitin-like modifications (such as SUMOylation [46,47,48] and Neddylation [49,50]), poly(ADP-ribosyl)ation(PARylation) [51,52], acetylation, and methylation [43,53,54]. These modifications regulate DSB recognition, damage signaling, and DNA repair by controlling the recruitment, stability, enzymatic activity, and protein interaction networks of DDR factors. Because NAD+ serves as the substrate for ADP-ribosyltransferases, cellular NAD+ availability may link metabolic state to ADP-ribosylation-dependent DNA damage signaling [52].

Previous reviews have summarized the biological functions and regulatory mechanisms of the classical PTMs involved in DDR regulation. However, the discovery of Kla, a newly recognized lysine acylation modification, has offered new insights into the link between cellular metabolic status and genome stability [55]. Lysine lactylation differs from classical lysine acylation, such as acetylation: it involves adding a lactyl group to lysine residues and is closely linked to cellular metabolic status [56]. Recent research has revealed the potential roles of lactylation in DDR. This modification may contribute to genome stability through several mechanisms, including regulating DNA damage signaling, DNA repair factor functions, and DNA repair efficiency [57,58]. Understanding how lactylation links cellular metabolism to DDR regulation may help explain how metabolic changes affect cellular responses to DNA damage. It may also provide new insights into potential targets for cancer therapy. Here, we review recent findings concerning the involvement of lysine lactylation in DDR signaling and DNA repair. We discuss its effects on key DNA repair factors and pathways, as well as its potential contribution to tumor progression and therapeutic resistance.

## 2. Lysine Lactylation

**Figure 1:**
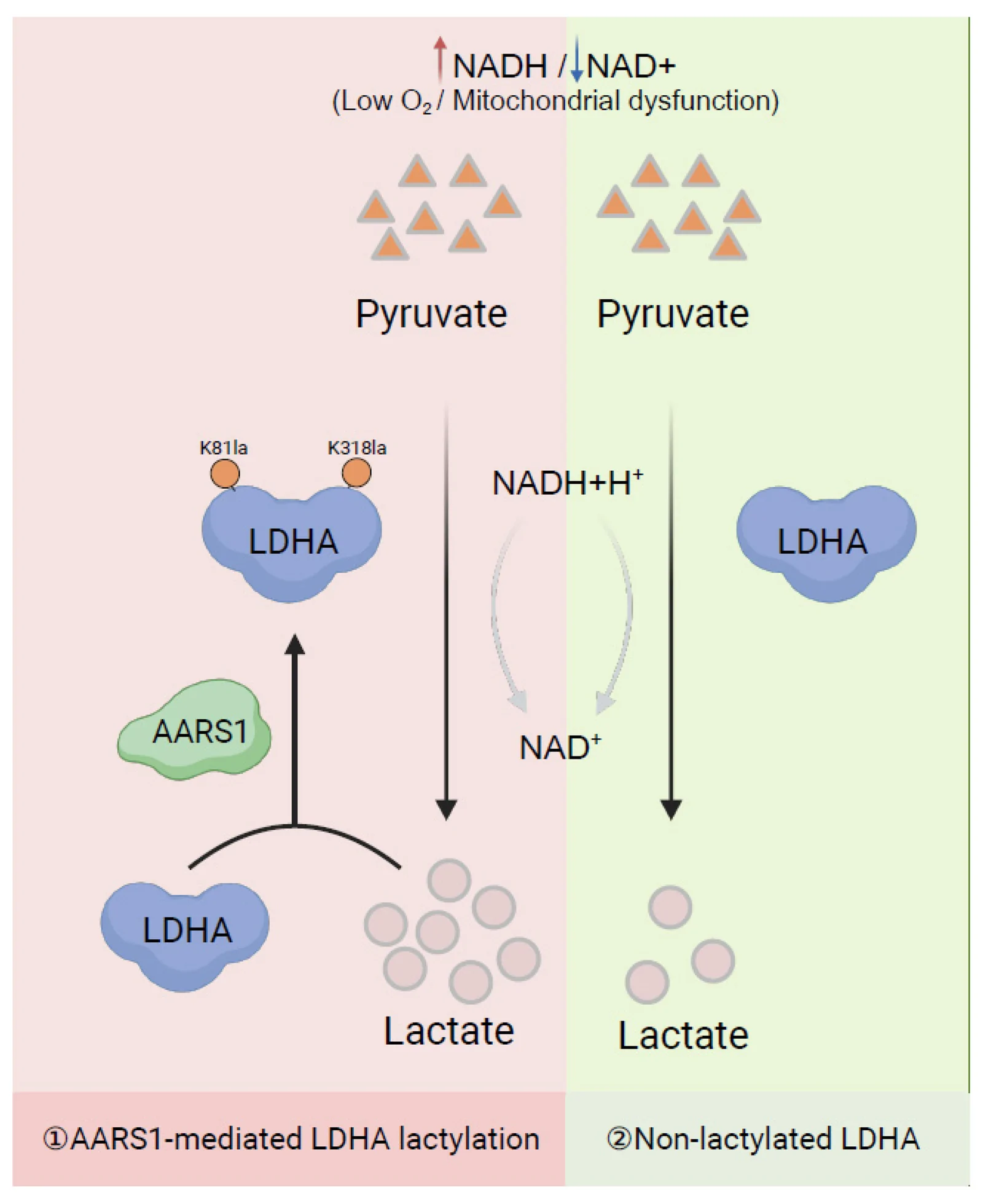
**AARS1-mediated LDHA lactylation links cellular metabolism to lactate production and protein lactylation.** Schematic illustration of the proposed relationship between cellular redox status, LDHA lactylation, and lactate production. The pink background (**①**) represents the condition in which AARS1-mediated LDHA lactylation is present, whereas the green background (**②**) represents the condition with reduced or absent LDHA lactylation. Under hypoxia or mitochondrial dysfunction, impaired NADH oxidation may increase the cytosolic NADH/NAD^+^ ratio, favoring the conversion of pyruvate to lactate by lactate dehydrogenase (LDH). AARS1-mediated lactylation of LDHA at lysine residues K81 and K318 (K81la and K318la, respectively) enhances its enzymatic activity, thereby promoting lactate production. This reaction consumes NADH and regenerates NAD^+^, supporting continued glycolysis. In contrast, non-lactylated LDHA can also catalyze the conversion of pyruvate to lactate, although the model depicts relatively lower lactate production. Increased lactate availability may contribute to protein lactylation through lactyl-CoA-dependent and other reported mechanisms. The proposed positive feedback relationship between LDHA lactylation, lactate accumulation, and global protein lactylation is illustrated. The increased NADH/NAD^+^ ratio represents a metabolic condition that favors lactate production and should not be interpreted as direct evidence of AARS1 activation. Abbreviations: AARS1, alanyl-tRNA synthetase 1; LDHA, lactate dehydrogenase A; LDH, lactate dehydrogenase; NAD^+^, oxidized nicotinamide adenine dinucleotide; NADH, reduced nicotinamide adenine dinucleotide; K81la and K318la, lactylation at lysine residues 81 and 318 of LDHA, respectively; PTM, post-translational modification.

Lactate was previously considered mainly a byproduct of anaerobic glycolysis and was associated with muscle fatigue and acidosis during exercise [59]. However, recent research has established that lactate also functions as a metabolite involved in energy metabolism and cellular signaling [59,60,61,62,63,64]. Glycolysis converts glucose to pyruvate through a series of enzymatic reactions [65]. Under aerobic conditions, pyruvate enters the mitochondria and is subsequently oxidized through the tricarboxylic acid (TCA) cycle [59,66,67]. The resulting reducing equivalents then support oxidative phosphorylation and ATP production [59,66,67]. The malate–aspartate and glycerol phosphate shuttles transport reducing equivalents derived from cytosolic NADH into mitochondria [68]. When oxygen is limited or mitochondrial function is impaired, NADH oxidation decreases, increasing the cytosolic NADH/NAD^+^ ratio [59]. Lactate dehydrogenase (LDH) catalyzes the conversion of pyruvate to lactate while regenerating NAD^+^, thereby allowing glycolysis to proceed [69]. Lactate metabolism is closely linked to protein lactylation. Lactate can serve as a metabolic precursor for lactyl-CoA formation, providing a source of lactyl groups for protein lactylation [70]. This pathway directly links cellular metabolic status to protein modification [56]. However, lactylation is not generated solely through lactyl-CoA-dependent mechanisms. Enzymatic and non-enzymatic pathways have been reported, suggesting that changes in lactate metabolism may affect Kla through multiple mechanisms (Figure 1).

### 2.1. Structure of Lactylation

**Figure 2:**
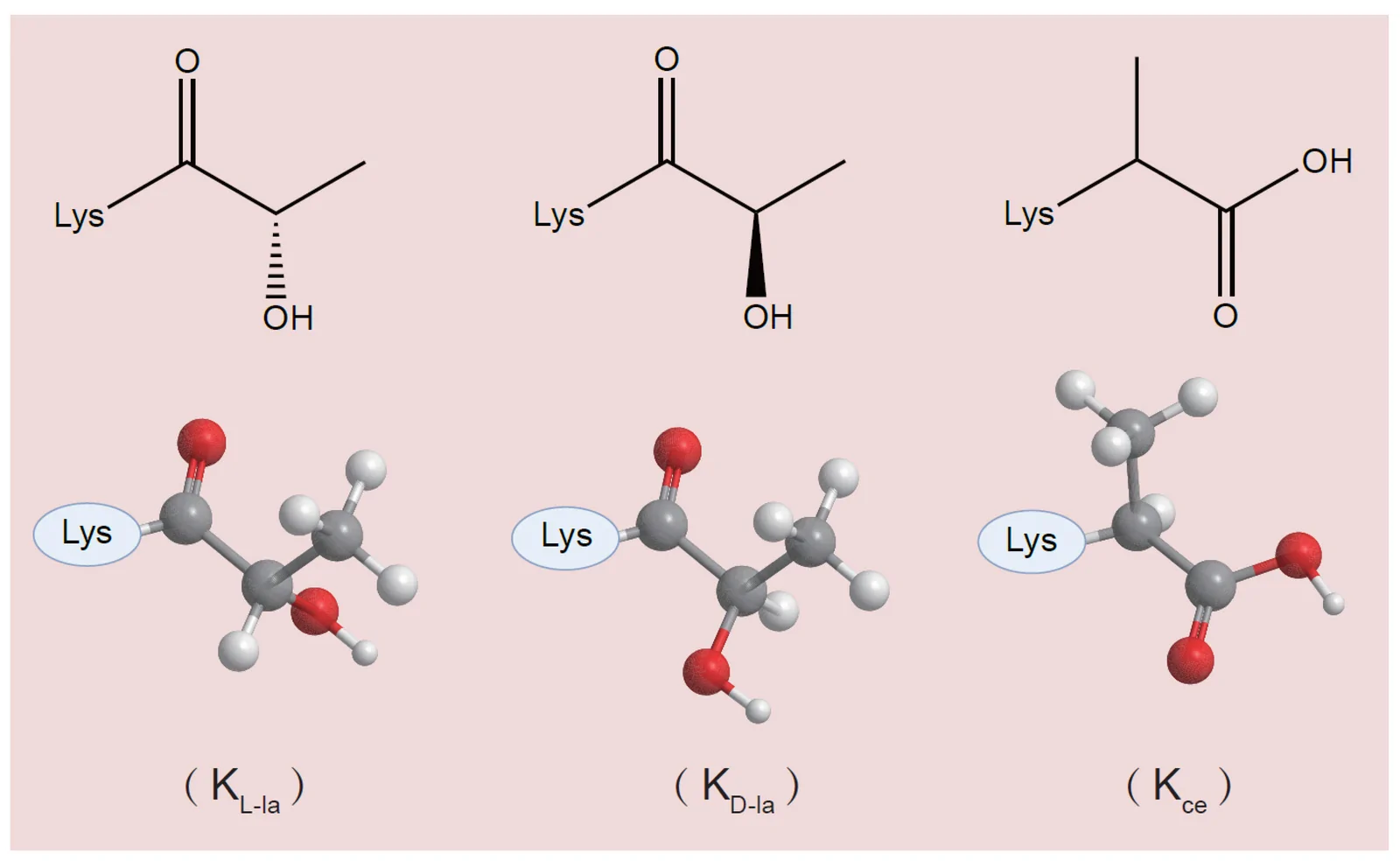
**Chemical structures of lysine lactylation-related modifications.** The three chemically distinct lysine modifications shown are L-lactyllysine (K_L-la), D-lactyllysine (K_D-la), and Nε-carboxyethyllysine (Kce). K_L-la and K_D-la are stereoisomeric forms of lactyllysine that differ in the stereochemical configuration of the lactyl group attached to the ε-amino group of lysine. In contrast, Kce is chemically distinct from lactyllysine and contains a carboxyethyl group attached to the ε-amino group of lysine. These modifications differ in their chemical structures and metabolic origins.

Lysine lactylation and classical acetylation are lysine acylation modifications, but they are structurally distinct. Using analytical chemistry and mass spectrometry, Zhao and colleagues identified three chemically distinct lysine modification isomers, L-lactyllysine (K_L-la), D-lactyllysine (K_D-la), and Nε-carboxyethyllysine (Kce), with K_L-la as the predominant lactylation isomer on histones [71]. These isomers differ in chemical structure and metabolic origin (Figure 2). K_L-la levels are dynamically influenced by glycolytic activity, including changes in glucose availability, whereas D-lactyllysine (K_D-la) and Nε-carboxyethyllysine (Kce) arise mainly through non-enzymatic reactions and are generally less abundant than K_L-la under glycolytic conditions [71,72].

L-lactylation is closely associated with L-lactate produced through glycolysis and may serve as an important link between cellular metabolism and protein regulation, particularly under the Warburg effect [55,71]. Among the lysine lactylation isomers currently characterized, L-lactylation has been the most extensively studied, and its molecular regulatory mechanisms and biological functions have been characterized. Importantly, several studies have shown that protein lactylation directly regulates DNA repair proteins, including MRE11 and NBS1, thereby promoting homologous recombination repair [57,58].

D-lactylation, associated with the glyoxalase pathway and D-lactate metabolism, represents an emerging lysine modification with regulatory mechanisms and biological functions that differ from L-lactylation; however, its specific biological roles remain to be fully elucidated [73]. In particular, it is largely unknown whether K_D-la directly contributes to DNA damage response and repair.

Nε-(Carboxyethyl)lysine is also of interest because it results from covalent modification of the ε-amino group of lysine residues by a carboxyethyl group(–CH_2_–CH_2_–COOH). This modification may result from a direct, non-enzymatic reaction between lysine residues and methylglyoxal, a highly reactive by-product of glycolysis [74,75]. Although Kce is chemically distinct from lactyllysine, its potential relevance to DNA damage and repair remains poorly characterized, and direct evidence that Kce regulates specific DNA damage response pathways is currently limited. These observations emphasize the need to clarify the biological functions of D-lactylation and carboxyethylation.

### 2.2. Identification of Lactylation

Lactylation is a newly recognized lysine acylation and post-translational modification (PTM), characterized by the covalent addition of a lactyl group to the ε-amino group of lysine residues [76]. This modification can affect several properties of target proteins, including stability [77,78], subcellular localization [78], protein–protein interactions [57,79], and transcriptional regulation [55]. Lactylation was first identified on histones and was initially associated with changes in chromatin structure and gene transcription [55]. Later research showed that lactylation also occurs in non-histone proteins, such as metabolic enzymes and signaling proteins. In these proteins, lactylation can regulate protein activity, stability, and molecular interactions, expanding its role beyond chromatin regulation to a range of cellular processes [58,77,78,80].

In 2019, Zhao et al. were the first to report lysine lactylation on histone lysine residues [55]. Their study identified 28 lactylation sites on core histones in human and mouse cells, including H3K18la, H3K23la, H3K27la, H4K8la, and H4K12la [55]. Among these sites, H3K18la was further characterized as a transcriptionally relevant histone lactylation marker [55]. Subsequent work showed that lactate produced through glycolysis contributes to histone lysine lactylation, thereby linking this modification to cellular metabolic states [55]. Unlike histone modifications, which mainly influence chromatin structure and accessibility, histone lactylation can modulate transcriptional activity at specific genomic loci [55]. In a macrophage polarization model of bacterial infection, histone lactylation increased during the late phase of M1 activation [55]. This increase was associated with enhanced expression of tissue-repair-related genes, including Arg1, which promoted the transition from a pro-inflammatory state toward a tissue-repair phenotype [55].

As studies on lactylation have advanced, increasing evidence shows that this modification is not limited to histones but is widespread among non-histone proteins [81,82]. In contrast to histone Kla, which mainly participates in chromatin-dependent transcriptional regulation, non-histone Kla can influence a broad range of biological processes by altering protein stability, enzymatic activity, subcellular localization, and molecular interactions [77,78,81]. Developments in mass spectrometry and chemical proteomics have increasingly facilitated the discovery of non-histone Kla sites in cellular compartments, including the nucleus, cytoplasm [82], and mitochondria [83,84], highlighting the diverse biological functions of non-histone Kla.

Although lactylation has been implicated in various physiological and pathological processes, its role in DNA repair remains unclear. In particular, the mechanisms by which metabolic changes regulate DNA repair through lactylation are not well understood. Recent evidence has linked lactate metabolism and protein lactylation to DNA repair regulation [80]. Li and colleagues showed that lactate dehydrogenase A (LDHA), a major enzyme responsible for lactate production, also regulates cellular protein lactylation [80]. Modifying LDHA at K81 and K318 enhances its enzymatic activity, increasing lactate production and global protein lactylation levels [80]. This finding suggests a positive feedback relationship between LDHA activity, lactate production, and protein lactylation [80]. Mass spectrometry and experimental validation identified lactylation on several DNA repair-related proteins, including FEN1, XRCC5, and XRCC6 [80]. LDHA-dependent modulation of protein lactylation was associated with enhanced formation of repair-associated protein complexes, such as FEN1–RAD1–RAD9A–HUS1 and XRCC5–XRCC6, thereby supporting DNA repair functions and contributing to cisplatin resistance in lung adenocarcinoma cells [80]. Together, these studies show that lactate functions not only as a signaling metabolite but also as a metabolic substrate for protein lactylation. They suggest that lactylation may link cellular metabolism with DNA repair and therapeutic resistance. The proposed relationship between LDHA lactylation, lactate production, and protein lactylation is illustrated in Figure 1.

### 2.3. Regulatory Machinery of Lysine Lactylation

**Figure 3:**
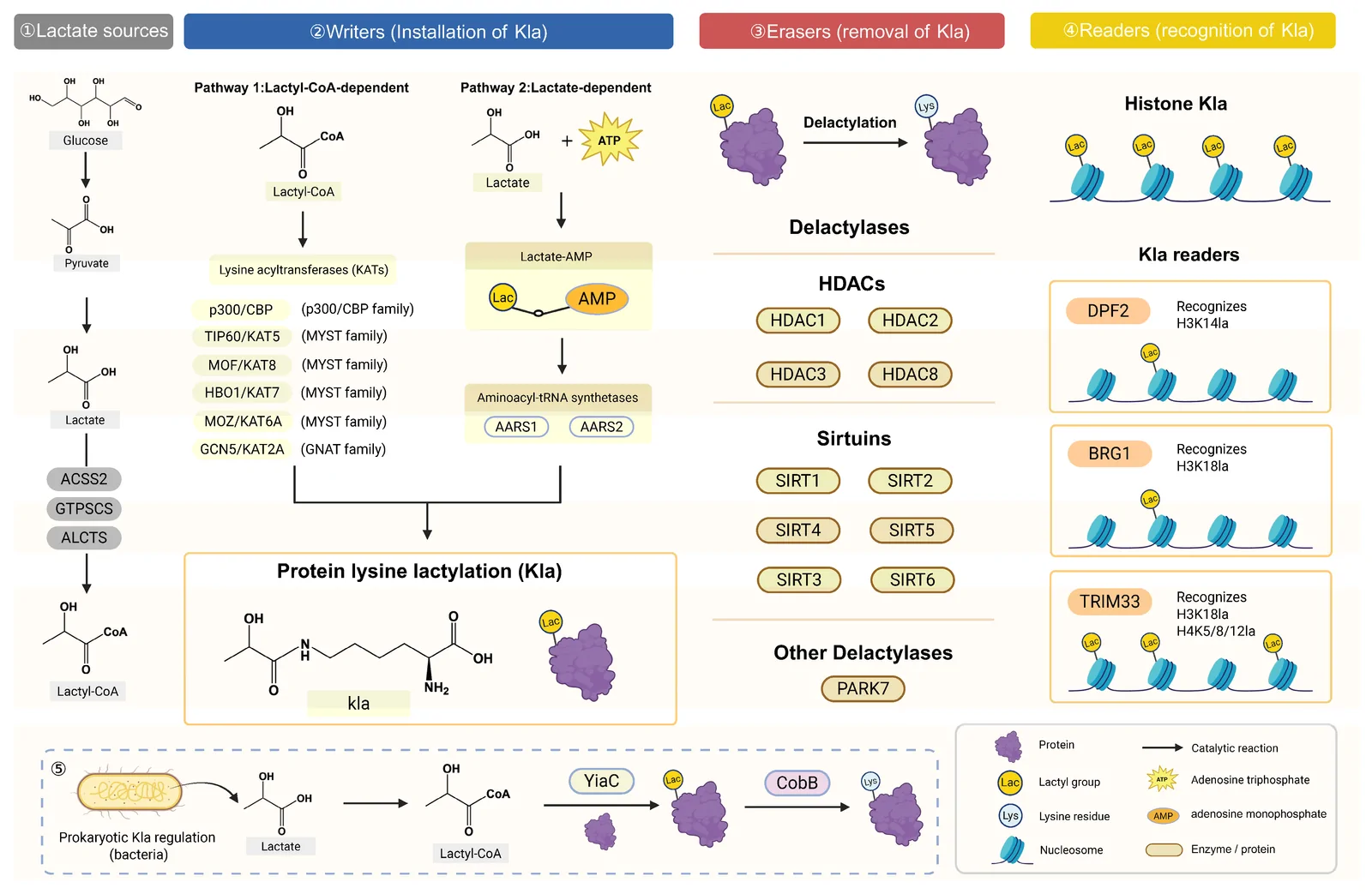
**Regulatory machinery of lysine lactylation.** Lysine lactylation is regulated by enzymatic writers, erasers, and reader proteins through distinct molecular mechanisms. In the lactyl-CoA-dependent pathway, lysine acyltransferases, including p300/CBP, KAT5/TIP60, KAT8/MOF, HBO1/KAT7, and KAT2A/GCN5, have been reported to catalyze the transfer of a lactyl group from lactyl-CoA to lysine residues. In the lactate-dependent pathway, AARS1 and AARS2 use L-lactate and ATP to generate a lactate-adenylate intermediate, followed by transfer of the lactyl group to lysine residues. Lactylation can be reversed by several deacylases, including HDAC1–3, HDAC8, SIRT1–3, and SIRT6, although their substrate and site specificities differ. Additional proteins, including SIRT4, SIRT5, and PARK7, have also been implicated in the regulation of protein lactylation, but their delactylase activities appear to be substrate- and context-dependent. Reported readers of histone lactylation include DPF2, BRG1, and TRIM33, which recognize specific lactylated histone residues and contribute to downstream transcriptional regulation. In bacteria, YiaC and CobB have been reported as a lactylation writer and delactylase, respectively. The figure summarizes currently characterized components of the lysine lactylation regulatory machinery and highlights the distinct enzymatic pathways involved in lactylation, delactylation, and recognition. **Abbreviations:** AARS1, alanyl-tRNA synthetase 1; AARS2, alanyl-tRNA synthetase 2; ATP, adenosine triphosphate; BRG1, Brahma-related gene 1; CBP, CREB-binding protein; DPF2, D4, zinc and double PHD fingers family 2; GCN5, general control non-derepressible 5; HBO1, histone acetyltransferase bound to ORC1; HDAC, histone deacetylase; KAT2A, lysine acetyltransferase 2A; KAT5, lysine acetyltransferase 5; KAT7, lysine acetyltransferase 7; KAT8, lysine acetyltransferase 8; MOF, males absent on the first; PARK7, Parkinsonism-associated deglycase; p300, E1A-binding protein p300; SIRT, sirtuin; TIP60, Tat-interactive protein 60 kDa; TRIM33, tripartite motif-containing protein 33.

Lysine lactylation is a reversible and dynamic protein post-translational modification; its regulation, as with many other PTMs, involves three functional groups: “writers”, which catalyze its addition [55]; “erasers”, which remove the modification [85]; and “readers”, which recognize specific lactylation markers and mediate their downstream effects [86]. Together, these regulatory components form a core regulatory framework through which lactylation signals are established, removed, and interpreted. Although lysine lactylation is distinct from conventional lysine acetylation, several enzymes involved in acetylation regulation, such as p300 and certain deacylases, have also been reported to mediate lactylation or delactylation [55,85,87] (Figure 3).

#### 2.3.1. Writers

Similar to many other PTMs, lysine lactylation is established through enzymatic and non-enzymatic mechanisms, with enzyme-catalyzed lactylation currently representing the major focus of investigations. Current evidence supports at least two distinct enzymatic mechanisms for lysine lactylation. The first mechanism is mediated by lysine acyltransferases, which transfer a lactyl group from lactyl-CoA to lysine residues [71,88]. The second depends on aminoacyl-tRNA synthetases (AARSs), which use L-lactate and ATP to form a lactate–adenylate intermediate before transferring the lactyl group to lysine residues [89,90]. The lactyl-CoA-dependent pathway is the best-characterized mechanism for several lysine acyltransferases. In 2019, Zhao and co-workers provided the first evidence that p300 can catalyze lactyl group transfer from lactyl-CoA to histones in vitro [55]. Based on these results, the authors proposed that p300 might function as a histone lactylation “writer”. Subsequent studies have provided further evidence supporting p300/CBP as important lactylation “writers”.

In 2023, Jia and colleagues reported that lysine residues K356 and K781 of the PIK3C3/VPS34 protein undergo lactylation, catalyzed by the acyltransferase KAT5/TIP60 [91]. This study further expanded the repertoire of lactylation “writer” enzymes beyond the previously identified p300/CBP family. In 2024, KAT8/MOF was reported to function as a lactyltransferase and pan-Kla “writer” [92]. eEF1A2 lactylation at K408 by KAT8 promotes translation elongation and protein synthesis, thereby supporting colorectal cancer growth, especially in a lactate-rich tumor microenvironment [92]. In 2024, Niu and colleagues identified HBO1 (KAT7/MYST2) as a lysine lactyltransferase and demonstrated that HBO1 directly catalyzes histone lactylation in vitro and intracellularly [88]. In 2025, Jia et al. further demonstrated that KAT7 positively regulates PRMT1 lactylation at K134 and K145 under hypoxic conditions [93]. KAT7 knockdown significantly attenuated hypoxia-induced PRMT1 lactylation, and this effect was reproduced using an independent shRNA targeting KAT7 [93]. This inhibitory effect was selective for wild-type PRMT1, whereas the PRMT1-2KR mutant (K134R/K145R) was unaffected [93]. Conversely, KAT7 overexpression increased lactylation of wild-type PRMT1 but had no such effect on the 2KR mutant [93]. Although functional experiments support KAT7’s role in promoting PRMT1 lactylation, Co-IP detected little interaction between KAT7 and PRMT1, leaving the question open of whether KAT7 directly catalyzes PRMT1 lactylation [93]. More recently, KAT2A (GCN5) was reported to function as a lactyltransferase in a lactyl-CoA-dependent manner [94]. Zhu and colleagues showed that ACSS2 acts as a lactyl-CoA synthetase and interacts with KAT2A to support KAT2A-mediated histone lactylation [94]. This pathway promotes histone lactylation and contributes to Wnt/β-catenin regulation and NF-κB signaling, thereby facilitating tumor immune evasion [94]. KAT6A/MOZ has also been implicated in lysine lactylation. Early evidence suggested that KAT6A could accommodate lactyl-CoA within its catalytic pocket, although these findings alone did not establish KAT6A as a lactyltransferase [88]. In 2026, Liu and colleagues provided cellular and functional evidence linking KAT6A to H3K9 lactylation in a PRCC-TFE3-rearranged renal cell carcinoma model [95]. Their study showed that PRCC-TFE3 fusion upregulates KAT6A and increases H3K9 lactylation, which promotes VEGFB transcription and activation of the VEGF-VEGFR2 signaling axis [95]. Collectively, these studies indicate that a growing number of enzymes, including canonical lysine acyltransferases such as p300/CBP, KAT5/TIP60, KAT8/MOF, KAT7/HBO1, and KAT2A/GCN5, KAT6A/MOZ, as well as non-canonical lactyltransferases such as AARS1, contribute to lysine lactylation, expanding the repertoire of enzymatic lactylation “writers”. However, the strength of evidence for direct catalytic activity varies between these enzymes.

Available evidence likewise suggests that different lactyltransferases may have distinct substrate profiles; however, the determinants of substrate selection remain incompletely understood. For example, HBO1/KAT7 has been shown to catalyze lactylation of multiple proteins, with most Kla sites regulated by HBO1 identified on histones. H3K9la also appears to be a prominent HBO1-regulated histone lactylation site [88]. However, it remains unclear whether individual lactyltransferases show a defined preference for histone or non-histone substrates. In addition, these enzymes’ activity and substrate selection may depend on the cellular context. For example, KAT5/TIP60-mediated lactylation of PIK3C3/VPS34 has been linked to nutrient deprivation and increased lactate production, whereas KAT8-mediated eEF1A2 lactylation has been associated with a lactate-rich tumor microenvironment [91,92]. These observations suggest that cellular metabolic states and specific stress conditions may influence lactyltransferase activity and substrate utilization; however, the extent and molecular basis of this context remain undetermined.

In addition to the lactyl-CoA-dependent pathway, AARSs have been implicated in an alternative lactate-dependent route of lysine lactylation [89,96]. In 2024, Zhou and colleagues identified AARS1 as an intracellular lactate sensor and lactyltransferase through CRISPRi screening [89]. They found that AARS1 directly binds lactate and catalyzes formation of lactate–AMP in an ATP-dependent manner, followed by transfer of the lactyl group to lysine residues. This mechanism contributes to global lysine lactylation, including lactylation of p53 at K120 and K139 [89]. In the same year, Li and colleagues reported that AARS1 and AARS2 function as intracellular L-lactate sensors and lactyltransferases in mammalian cells [96]. Their study showed that AARS1/2 directly catalyzes ATP-dependent lactylation using L-lactate as the substrate, without requiring lactyl-CoA. In particular, AARS2 was shown to mediate cGAS lactylation and thereby regulate cGAS activity [96]. These findings provide evidence for a lactate-dependent mechanism of lysine lactylation that is distinct from lactyl-CoA-dependent pathways and expand the known enzymatic machinery involved in lactylation.

The studies discussed above indicate that lysine lactylation can be established through distinct enzymatic mechanisms. Lactate serves as an important metabolic substrate for lysine lactylation, although intracellular lactate abundance does not necessarily translate into a proportional increase in Kla. Kla formation is also influenced by the availability of lactyl-donor intermediates and the enzymes involved in its formation [97,98]. Exogenous lactate treatment has been widely used to induce lactylation; for example, treatment with 10 mM lactate was reported to increase histone Kla levels [55,99], while concentrations as high as 25 mM have also been used in macrophage studies [55,100]. Therefore, these experimental concentrations should not be interpreted as defining a universal lactate threshold for lysine lactylation, because the relationship between lactate availability and Kla formation depends on the cellular context and the enzymatic or non-enzymatic pathway involved [55,97].

Lactate concentrations in healthy tissues and extracellular fluids are generally in the low millimolar range and can vary substantially with physiological and metabolic states [101,102]. In some tumor models, substantially higher lactate concentrations have been detected in the tumor microenvironment; for example, lactate concentrations of approximately 10–11 mM have been measured in tumor-conditioned medium and tumor interstitial fluid, respectively [103]. However, these values do not represent all tumors, as lactate concentrations can vary considerably across tumor types, anatomical regions, metabolic states, and measurement compartments [102,104]. Accordingly, the millimolar lactate concentrations commonly used in cell culture experiments may model aspects of lactate-rich metabolic environments, but their physiological relevance should be interpreted with caution. In particular, extracellular lactate concentrations in vitro do not necessarily recapitulate the dynamic and spatially heterogeneous lactate exposure experienced by cells in vivo [102,104].

Lactate-mediated Kla regulation also depends on the substrate utilization mechanisms and catalytic properties of the enzymes involved. In the AARS1/2-mediated pathway, ATP activates lactate to form a lactyl-AMP intermediate, which is transferred to lysine residues on target proteins [83,105]. In contrast, lactyl-CoA-dependent pathways require lactyl-CoA generation, which serves as the acyl donor for lysine acyltransferases such as p300 and KAT2A [94,106]. These enzymes have different substrate-binding properties and catalytic efficiencies, and binding affinity alone cannot determine their catalytic performance under cellular conditions. For example, AARS1 binds lactate with a dissociation constant *K*_D_ of 2.06 μM [105], whereas AARS2 exhibits a *K*_m_ of 5.21 mM for lactate in its lactyltransferase reaction using a synthetic PDHA1 K336-containing peptide as the protein substrate [83]. These parameters describe different aspects of enzyme–substrate interactions and should therefore not be interpreted as directly equivalent measures. Kinetic characterization of GTPSCS likewise indicates that its lactyl-CoA synthetase activity depends on lactate availability, with a reported *K*_m_ of 15.32 ± 1.28 mM for lactate [106]. Conversely, KAT2A has been reported to use lactyl-CoA as a substrate with a *K*_m_ of approximately 0.49 μM [34,94]. Because these kinetic parameters were obtained for different enzymes, reactions, substrates, and experimental systems, they should not be used as a unified measure of cellular lactate sensitivity. Taken together, these observations indicate that substrate-binding affinity and catalytic efficiency are distinct biochemical properties, and that intracellular lactate concentration alone is therefore insufficient to predict cellular Kla levels.

The local production of lactylation substrates and their subcellular distribution may also contribute to Kla regulation. Functional coupling between ACSS2 and KAT2A has been reported: nuclear ACSS2 converts lactate to lactyl-CoA and interacts with KAT2A, which uses lactyl-CoA for histone lactylation [94]. Similarly, nuclear GTPSCS has been shown to interact with p300 and generate lactyl-CoA in the nucleus, thereby promoting histone lactylation [106]. These findings suggest that lactyl-CoA may be generated and utilized locally rather than determined solely by a uniform intracellular metabolite pool. Thus, measurements of total intracellular lactate or lactyl-CoA concentrations may not fully reflect the substrate availability experienced by individual enzymes or lysine sites.

The stoichiometric occupancy of Kla remains another important question. Although many studies have reported changes in global or site-specific Kla following lactate treatment, metabolic alterations, or manipulation of lactyltransferase activity, the absolute occupancy of individual lysine sites has not yet been systematically characterized across different cell types and physiological or pathological conditions [107,108]. A recent quantitative proteomic study of histones from mouse testes estimated low lactylation stoichiometry at multiple H3 and H4 lysine residues, with Kla levels ranging from approximately 0.01% to 0.44% across the sites examined [108]. However, these measurements were obtained from mouse testis histones and therefore cannot be directly extrapolated to other tissues, cell types, or disease models [108]. Further studies using stable isotope-labeled standards and targeted quantitative mass spectrometry are important for determining Kla’s absolute occupancy and its dynamic changes under defined metabolic conditions. Overall, lactate should be regarded as an important metabolic input for Kla regulation rather than a direct quantitative measure of Kla abundance. The extent of lactylation and its biological consequences are likely determined by the combined effects of substrate activation, enzyme kinetics, subcellular localization, and site-specific regulatory mechanisms.

#### 2.3.2. Erasers

Protein lactylation can be dynamically reversed by delactylases, which function as “erasers” of this modification. Most characterized delactylases belong to the deacylase family and remove lactyl groups from lysine residues through enzymatic deacylation reactions, thereby regulating the functional state of target proteins.

In 2022, Moreno-Yruela and colleagues systematically screened all 18 mammalian lysine deacylases for delactylase activity and demonstrated that class I HDACs (HDAC1–3) exhibit substantial delactylase activity toward lysine lactylation in vitro [85]. Their results showed that class I HDACs (HDAC1–3) displayed substantial delactylase activity in vitro [85]. Although all three enzymes exhibited activity, HDAC3 showed the highest efficiency in removing L- and D-lactyllysines under the conditions tested [85]. The same study showed that SIRT1–3 also possess L-lactyllysine delactylase activity toward histone lactylation in vitro, including specific sites such as H3K18la and H4K5la [85].

HDAC1–3, which belong to the class I HDAC family, regulate histone acetylation alongside other lysine acylation modifications, including crotonylation (Kcr) [109,110] and lysine β-hydroxybutyrylation [111]. Consistent with this broader substrate specificity, HDAC2 has been identified as a delactylase for the METTL3 protein in triple-negative breast cancer, where it directly removes lactylation from lysine 27 of METTL3 [112]. Several studies have shown that HDAC2 overexpression reduces overall protein lactylation levels, including histone H3K18 lactylation [63]. HDAC8, another member of the same family, functions as a delactylase of PRMT1 [93]. HDAC8 knockdown increased PRMT1 lactylation, confirmed by multiple independent shRNAs in HeLa and MDA-MB-231 cells [93]. In contrast, HDAC8 overexpression reduced lactylation of wild-type PRMT1 but did not affect the 2KR mutant (K134R/K145R), indicating that HDAC8 specifically regulates PRMT1 lactylation at K134 and K145 [93]. Hypoxia downregulates HDAC8, thereby relieving its delactylation of PRMT1 and increasing PRMT1 lactylation at K134 and K145, which subsequently promotes asymmetric dimethylation (aDMA) of vimentin at R64 [93].

SIRT1–3 are core members of the sirtuin family and have been implicated in protein lactylation regulation [87]. Wang and colleagues demonstrated that SIRT3 regulates lactylation levels in hepatocellular carcinoma cells by comparing wild-type SIRT3 with the loss-of-function mutant SIRT3-H248Y in Huh-7 cells. They also investigated the experimental manipulation of cellular lactate levels [113]. Sun and colleagues screened SIRT1–7 for their ability to regulate α-MHC K1897 lactylation and identified SIRT1 as the delactylase responsible for this modification [114]. Using crystal structure analysis and complementary biochemical approaches, including NAD^+^ consumption and recycling assays, isothermal titration calorimetry, and HPLC–MS, Fan and colleagues demonstrated that SIRT3 exhibits strong delactylase activity toward H4K16la [115]. Notably, sirtuin family’s delactylase activity exhibits substrate and site dependence. SIRT1 has been reported to remove lactyl groups from several histone sites, including H3K18la and H4K5la [85], and regulates lactylation on non-histone proteins, such as delactylation of α-MHC at K1897 [114]. Thus, current evidence does not support a simple preference of SIRT1 for either histone or non-histone substrates. Other sirtuin family members show similar site-dependent features. SIRT2 exhibits delactylase activity toward multiple histone sites; however, this is markedly reduced when the lactylated lysine is immediately adjacent to glycine or proline residues [116]. SIRT3 also shows relatively high delactylation activity toward H4K16la and contributes to the delactylation of mitochondrial non-histone proteins, including PDHA1 and CPT2 [83,115]. Together, these findings suggest that the substrate and site specificity of sirtuin delactylases may be influenced by the substrate identity and local sequence context surrounding the lactylated lysine.

More recent studies have identified SIRT6 as another delactylase, expanding the known repertoire of lactylation “erasers”. In 2024, Li and colleagues reported that SIRT6 acts as a delactylase by directly binding to ALKBH5 and catalyzing the removal of the lactyl group from K284, thereby influencing ALKBH5-dependent antiviral innate immune responses against DNA viruses such as HSV-1, KSHV, and mpox virus [117]. More recently, Xie and colleagues identified SIRT6 as a delactylase for TANK-binding kinase 1 (TBK1) and revealed an AARS1–SIRT6 regulatory axis that dynamically controls TBK1 lactylation during antiviral responses [118]. Together, these findings support SIRT6 as an emerging delactylase that can regulate distinct non-histone substrates in different cellular contexts, expanding the substrate and functional landscape of lactylation erasers.

Evidence for the delactylase activities of SIRT4 and SIRT5 remains limited and appears to depend on the experimental context. In 2023, Mao and colleagues showed in a biochemical study that SIRT3 can remove lactyl groups from PDHA1 K336 and CPT2 K457/K458, whereas neither SIRT4 nor SIRT5 exhibited detectable delactylase activity toward the tested mitochondrial lactylated substrates [83]. More recent studies, however, have implicated SIRT4 and SIRT5 in regulating lactylation on specific proteins, including STING and CKB, respectively [119,120]. In 2026, Sun and colleagues identified PARK7 as a novel delactylase [121]. Using lactylome profiling combined with immunoprecipitation–mass spectrometry, Sun and colleagues reported that PARK7 exhibits delactylase activity and catalyzes the removal of the lactyl group from lysine 280 (K280) of the RNA splicing-related protein SF3B2 [121]. PARK7-mediated delactylation of SF3B2 suppresses tumor growth through an immune-dependent mechanism [121].

Importantly, lactylation regulation is not restricted to eukaryotic systems, as corresponding enzymatic mechanisms have also been identified in prokaryotes. In 2022, Dong and colleagues first reported the Kla regulatory system in prokaryotes [122]. Using a pan-Kla antibody, the study detected widespread lysine lactylation in bacterial proteins and identified two enzymes involved in its regulation: YiaC as a lactylation “writer” and CobB as a “delactylase” [122]. Both in vitro and in cells, YiaC and CobB catalyze the addition and removal of lysine lactylation, respectively, thereby modulating metabolic enzyme activity [122].

#### 2.3.3. Readers

“Reader” proteins containing specific recognition domains have been identified as mediators of lactylation-dependent molecular responses. Reported readers of histone lactylation include DPF2, BRG1, and TRIM33. In 2024, Zhai and colleagues combined multivalent photo-crosslinking probe technology with quantitative proteomic screening to identify DPF2 as a specific reader of histone H3K14 lactylation (H3K14la) [86]. Using in vitro binding assays, immunoprecipitation, and chromatin profiling with CUT&Tag technology, they demonstrated that DPF2 specifically recognizes H3K14la and significantly colocalizes with this modification at multiple oncogene promoter regions [86].

Also in 2024, another study shed light on two molecular mechanisms by which Dux proteins control cellular reprogramming and showed that the chromatin-remodeling factor BRG1 interacts with H3K18la [123]. This recognition promotes enrichment of H3K18la at the promoters of key mesenchymal-to-epithelial transition (MET) genes [123]. It provides a mechanism through which lactylation regulates transcription [123]. Around the same time, AlphaScreen-based screening of 28 human bromodomain-containing proteins identified TRIM33 as the only protein tested that could bind histone Kla. TRIM33 showed sequence-specific recognition of H3K18la and H4K5/8/12la [124].

These studies provide evidence that reader proteins can directly reorganize lactylation, particularly in the context of histone modification. However, whether similar reader-mediated mechanisms operate on lactylated DNA damage response proteins remains largely unknown. Lactylated DDR factors identified to date include MRE11, NBS1, BLM, XLF, and XRCC1. They have been implicated in DNA repair through distinct mechanisms, including altered DNA-binding activity, protein–protein interactions, protein stability, and subcellular localization [57,58,125,126,127]. To date, no well-established evidence has demonstrated that a dedicated lactylation reader directly recognizes these modified DDR proteins and mediates their repair-related functions. Thus, a direct reader-mediated mechanism linking lactylation to the regulation of lactylated DDR proteins has not yet been established. Identifying DDR-associated lactylation readers and determining how they recognize specific lactylated repair factors represent important areas for future investigation.

## 3. Role of Lactylation in DNA Damage Response

DNA damage refers to changes in the structure or sequence of cellular DNA and occurs across a wide range of organisms [128]. It can affect normal cellular functions and contribute to cellular senescence, tumorigenesis, and other pathological processes [129]. At the same time, DNA damage activates cellular repair pathways that remove or repair damaged DNA and help maintain genome stability [129]. DNA lesions can be induced by a range of factors, including chemical agents, ultraviolet (UV) radiation, and ionizing radiation (IR) [130]. When DNA damage is inadequately repaired, mutations may accumulate, increasing the risk of cancer initiation and progression [131,132]. Among these lesions, DSBs are particularly detrimental because they can cause chromosome fragmentation and rearrangements. Failure to resolve DSBs can contribute to genomic instability and, in severe cases, result in cell death [1,133].

**Figure 4:**
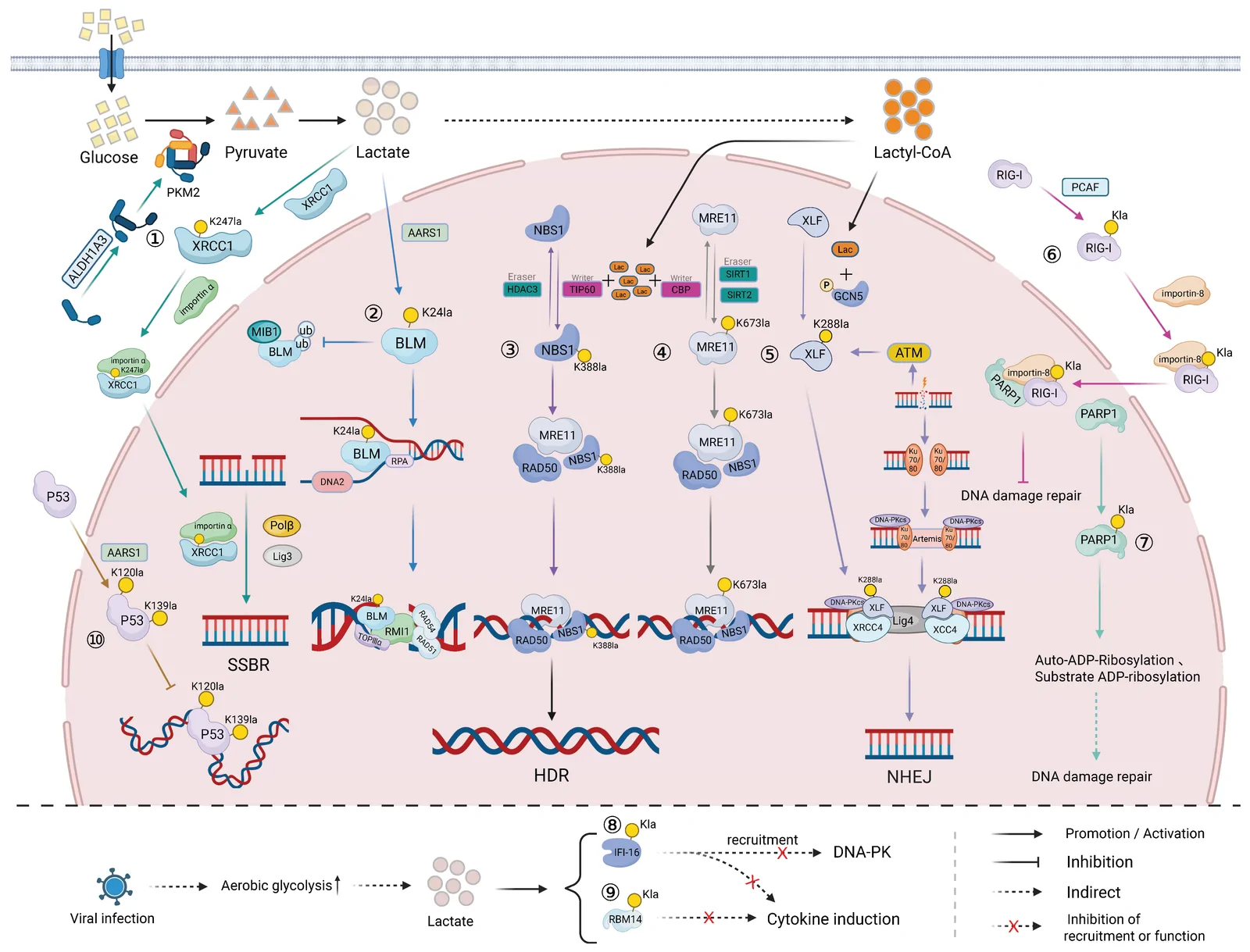
**Lactylation regulates the DNA damage response and DNA repair.** Increased cellular lactate levels can promote lysine lactylation (Kla) of multiple proteins involved in the DNA damage response (DDR), DNA repair, and antiviral signaling. During homologous recombination (HR), AARS1-mediated BLM K24 lactylation increases BLM stability by reducing MIB1-dependent ubiquitination and degradation, thereby promoting DNA end resection and HR repair. CBP-mediated MRE11 K673 lactylation increases the DNA-binding capacity of MRE11 and facilitates DSB end resection. TIP60-mediated NBS1 K388 lactylation promotes the interaction between NBS1 and MRE11, facilitating MRN complex assembly and recruitment to DSB sites. During non-homologous end joining (NHEJ), DNA damage-induced ATM-dependent phosphorylation of GCN5 enhances its interaction with XLF and promotes XLF K288 lactylation. XLF K288 lactylation strengthens the interaction between XLF and Ku80 and promotes NHEJ repair. Lactate-induced XRCC1 K247 lactylation enhances its interaction with importin α and promotes nuclear localization, thereby facilitating DNA repair. Lactylation S1. mediated p53 K120 and K139 lactylation impairs p53 DNA binding and transcriptional activity, thereby linking lactate metabolism to p53-dependent cellular responses. PARP1 lactylation within its automodification domain can enhance PARP1 ADP-ribosylation activity by counteracting acetylation at overlapping lysine residues; however, direct evidence that this modification promotes DNA repair remains limited. In contrast, PCAF-mediated RIG-I (DDX58) lactylation promotes its nuclear translocation and interaction with PARP1, which inhibits PARP1 activity and impairs DNA repair. During herpesvirus infection, IFI16 K90 lactylation reduces the recruitment of active DNA-PK to viral DNA and attenuates antiviral signaling, although the precise molecular mechanism underlying this effect remains unclear. RBM14 lactylation may also alter antiviral signaling. Solid arrows indicate experimentally supported promotion or activation; dashed arrows indicate indirect or incompletely defined effects; red blunt-ended lines indicate inhibition. The bacterial lactylation regulatory pathway is not included because it is outside the scope of the DNA damage response mechanisms discussed in the main text. **Abbreviations:** AARS1, alanyl-tRNA synthetase 1; ATM, ataxia-telangiectasia mutated; BLM, Bloom syndrome protein; CBP, CREB-binding protein; DSB, DNA double-strand break; DDR, DNA damage response; DNA-PK, DNA-dependent protein kinase; GCN5, general control non-repressible 5; HDAC3, histone deacetylase 3; HR, homologous recombination; IFI16, interferon gamma inducible protein 16; Kla, lysine lactylation; MIB1, mindbomb E3 ubiquitin protein ligase 1; MRN, MRE11–RAD50–NBS1 complex; NBS1, Nijmegen breakage syndrome protein 1; NHEJ, non-homologous end joining; PARP1, poly(ADP-ribose) polymerase 1; PCAF, p300/CBP-associated factor; RIG-I, retinoic acid-inducible gene I; TIP60, Tat-interactive protein 60 kDa; XRCC1, X-ray repair cross-complementing protein 1; XLF, XRCC4-like factor.

Lactylation, an emerging protein post-translational modification, has recently been identified as a potential regulatory mechanism in the DDR [58,134]. By modulating the activity and DNA-binding properties of DNA damage response factors, lactylation may influence DNA damage recognition and subsequent repair responses [58]. Currently identified mechanisms by which lactylation regulates DNA damage signaling and repair are summarized in Figure 4 and Table 1.

### 3.1. The Role of Lactylation in the MRN Complex During Homologous Recombination Repair

Following DSB formation, the MRN complex acts as an early sensor of DNA damage and plays essential roles in DSB recognition, ATM activation, and initiation of DNA repair [26]. The complex comprises MRE11, RAD50, and Nijmegen breakage syndrome protein 1 (NBS1, also known as nibrin), with two copies of each subunit forming a heterohexamer [136].

The MRN complex rapidly detects and binds to DNA ends produced by DSBs [137]. The MRN complex recruits and activates ATM kinase to initiate downstream phosphorylation signaling and cell-cycle checkpoint responses; by contrast, MRE11 nuclease activity contributes directly to DNA-end processing required for subsequent HDR repair [136,138].

#### 3.1.1. MRE11 Lactylation Regulates Homologous Recombination Repair

As the catalytic core of the MRN complex, MRE11 exhibits Mn^2+^-dependent 3′–5′ exonuclease and endonuclease activities that participate in DNA end processing and resection after DSB formation [136,139,140]. Recent evidence shows that MRE11 is subject to lactylation, and this modification regulates homologous recombination repair [58]. Through integrated analyses of TCGA datasets, lactylome profiling, and cellular functional assays, researchers identified multiple lactylation sites on MRE11; among these, K673 was identified as a major DNA damage-induced lactylation site [58]. MRE11 K673 lactylation markedly increases MRE11’s DNA-binding capacity, thereby facilitating DSB end resection and HDR repair [58]. Further investigations have elucidated the regulatory mechanisms underlying MRE11 lactylation. Among these regulators, CREB-binding protein (CBP) functions as a lactyltransferase that promotes MRE11 lactylation, whereas SIRT1 and SIRT2 act as delactylases that remove lactyl groups from MRE11, thereby regulating its lactylation status [58]. Furthermore, ATM-mediated phosphorylation of CBP is required for MRE11 lactylation [58] (Figure 4) ④. Together, these findings provide mechanistic evidence that lactylation connects cellular metabolism to HDR repair. They also suggest that metabolic reprogramming may alter DNA repair capacity and contribute to tumor therapeutic resistance.

#### 3.1.2. NBS1 Lactylation Promotes MRN Complex Assembly and Homologous Recombination Repair

NBS1 is an essential component of the MRN complex and functions in the early stages of DSB response. It helps recruit and retain the MRN complex at DNA damage sites. Previous studies have shown that NBS1 is required for efficient localization of the MRN complex to DNA breaks [141,142]. Loss of NBS1 reduces the accumulation of MRE11 and RAD50 at DSB sites, suggesting that NBS1 helps maintain the MRN complex at damaged DNA [143,144]. Subsequent studies revealed that NBS1 lactylation also contributes to regulating MRN complex function [57]. 4D proteomic analysis identified NBS1 as a lactylation substrate and revealed that NBS1-K388 lactylation was significantly increased in drug-resistant tumor cells [57]. NBS1 K388 lactylation is catalyzed by TIP60 and removed by HDAC3; its levels are further increased by lactate accumulation and cisplatin treatment [57]. Mechanistic studies showed that NBS1-K388 lactylation induces a conformational change in NBS1, promoting its interaction with MRE11 and enhancing MRN complex assembly and recruitment to DSB sites [57]. The enhanced recruitment of the MRN complex subsequently facilitates HDR repair and increases tumor cell tolerance to DNA-damaging agents [57]. Moreover, LDHA contributed to the increased lactate production observed in drug-resistant tumor cells. Genetic depletion or pharmacological inhibition of LDHA reduced NBS1 K388 lactylation, impaired DNA repair, and increased sensitivity to DNA-damaging treatment [57]. Importantly, the available evidence supports a model in which LDHA-dependent lactate production promotes NBS1 K388 lactylation through TIP60, rather than indicating that LDHA itself acts as the NBS1 lactyltransferase [57]. Consistent with this mechanism, analysis of gastric cancer samples from patients receiving neoadjuvant chemotherapy showed that higher LDHA expression was positively associated with NBS1 lactylation and correlated with poorer patient outcomes [57]. These findings suggest that the LDHA–lactate–TIP60–NBS1 lactylation axis may constitute a metabolic–post-translational regulatory mechanism linked to tumor chemoresistance [57] (Figure 4, ③).

### 3.2. BLM Lactylation Promotes Homologous Recombination Repair

DNA helicases are ATP-dependent enzymes that use energy from ATP hydrolysis to remodel DNA structures [145]. BLM, a member of the RecQ helicase family, plays an important role in DNA replication, the DNA damage response, and homologous recombination repair [146,147,148]. BLM promotes DNA end resection and the generation of single-stranded DNA intermediates [149,150]. It also regulates RAD51-dependent homologous recombination and contributes to several stages of HDR repair [149,150].

Lactylome analyses have identified numerous DNA repair-associated proteins, many of which are enriched in DNA repair-related pathways. Recent work has identified BLM as a substrate of AARS1-mediated lactylation and shown that lactylation at K24 promotes BLM stability and DNA repair activity [125]. In this study, AARS1 knockdown reduced BLM lactylation, whereas AARS1 overexpression increased it, and AARS1 was found to interact with BLM [125]. Treatment with 20 mM sodium lactate also increased BLM-K24 lactylation in cultured cells [125]. However, this concentration was used as an experimental treatment and should not be interpreted as a physiological or intracellular threshold for BLM lactylation. The study did not establish a quantitative concentration–response relationship between intracellular lactate levels and BLM-K24 lactylation.

Lactylation of BLM at K24 suppresses MIB1-dependent ubiquitination and degradation. This results in greater BLM stability and stronger interactions with DNA repair proteins, such as DNA2, TOP2A, RPA, and RAD54, thereby facilitating HDR-mediated DSB repair [125]. The BLM-K24R mutant, which prevents lactylation at this site, showed reduced HR repair and DNA end resection, whereas lactate treatment enhanced wild-type BLM function but had little effect on the K24R mutant [125]. Moreover, increased AARS1 expression in anthracycline-resistant tumor cells was associated with increased BLM-K24 lactylation, enhanced DNA repair capacity, and chemoresistance [125]. Together, these findings support an AARS1–BLM-K24 lactylation pathway in HR repair regulation and anthracycline resistance, and the quantitative relationship between intracellular lactate availability and BLM-K24 lactylation remains to be determined (Figure 4, ②).

### 3.3. XLF Lactylation Promotes Non-Homologous End Joining Repair

NHEJ begins when the Ku70/Ku80 heterodimer recognizes broken DNA ends and subsequently recruits DNA-PKcs and other repair factors to the damaged sites [37,151]. XLF (XRCC4-like factor) is a key component of the NHEJ machinery [152]. XLF shares structural similarities with XRCC4 and promotes DNA end bridging and repair complex assembly, which are important for efficient DNA ligation [152,153]. Recent evidence indicates that lactylation of XLF influences the efficiency of NHEJ-mediated repair [126]. Following DSB formation, ATM-dependent phosphorylation of GCN5 enhances the interaction between GCN5 and XLF, thereby promoting XLF K288 lactylation [126]. XLF-K288 lactylation strengthens the interaction between XLF and Ku80 and induces a conformational change in the Ku80 vWA domain [126]. This change increases the stability of the Ku-XLF complex and promotes NHEJ repair [126]. SIRT1 is a delactylase for XLF. It removes the K288 lactyl group, providing a mechanism for dynamic control of XLF lactylation during NHEJ repair [126] (Figure 4, ⑤).

### 3.4. Dual Regulatory Roles of Lactylation in Antiviral Immune Evasion and the DNA Damage Response

Recent studies have shown that HCMV and HSV-1 infections induce lysine lactylation of host and viral proteins, thereby suppressing innate antiviral immune responses [135]. Notably, lactylation of IFI16 at K90 impairs recruitment of active DNA-PK to viral DNA [135]. Experimental evidence showed that the K90 lactylation mimic K90Q did not substantially affect IFI16 expression, nuclear localization, or its localization near incoming viral DNA. However, compared with WT and K90R IFI16, K90Q markedly reduced the enrichment of phosphorylated DNA-PK at S2056 (pDNA-PK) in ICP4-positive viral transcription compartments [135]. These findings indicate that K90 lactylation does not primarily impair IFI16 localization to viral DNA but instead interferes with the subsequent recruitment of active DNA-PK, thereby disrupting IFI16-mediated antiviral signaling. The precise molecular basis by which K90 lactylation interferes with DNA-PK recruitment has not yet been determined [135]. This modification not only impairs IFI16-mediated inhibition of viral gene expression but also significantly reduces cytokine production in host cells [135]. Thus, K90 lactylation provides a mechanism by which herpesvirus-induced metabolic remodeling attenuates the antiviral function of the IFI16–DNA-PK axis [135]. These findings suggest that lactylation can exert context-dependent effects on DDR-associated signaling, suppressing antiviral signaling through IFI16–DNA-PK while promoting DNA repair through lactylation of specific repair factors [57,58,135]. This study further supports AARS1’s role as a lactyltransferase and shows that lactylation sites are enriched in intrinsically disordered regions (IDRs) during viral infection [96,135] (Figure 4, ⑧). Moreover, RBM14 K600 lactylation promotes HCMV spread and suppresses innate antiviral signaling, whereas IFI16 K90 lactylation impairs the recruitment of active DNA-PK to viral DNA, thereby promoting herpesvirus immune evasion [135] (Figure 4, ⑨).

### 3.5. p53 Lactylation Links Metabolic Reprogramming to DNA Damage Response and Cell Fate Regulation

p53 is a major tumor suppressor and transcription factor that regulates DNA repair, cell-cycle progression, and other cellular processes [154,155]. By activating a range of target genes, p53 helps prevent malignant transformation, and its activity is also regulated by post-translational modifications, although the underlying mechanisms remain incompletely understood [154,156]. In 2024, Zong and colleagues reported that tumor-derived lactate acts as a natural inhibitor of p53 by promoting its lactylation [89]. Using a genome-wide CRISPR screen, Zong and colleagues identified alanyl-tRNA synthetase 1 (AARS1) as an intracellular lactate sensor and lactyltransferase [89]. Proteomic analysis identified numerous AARS1 targets, including p53, and further experiments established the mechanism by which AARS1-mediated p53 lactylation promotes tumorigenesis [89]. AARS1-mediated lactylation of p53 at K120 and K139 within the DNA-binding domain (DBD) impairs DNA binding, reduces liquid–liquid phase separation, and suppresses transcriptional activity, thereby promoting tumorigenesis [89]. However, the role of p53 lactylation in specific DNA repair processes remains unclear and requires further investigation. This study identified a new mechanism linking the metabolite lactate to p53 function through lactylation and suggested a potential therapeutic avenue for cancer treatment (Figure 4, ⑩).

### 3.6. Molecular Mechanisms Linking Lactylation to the DNA Damage Response Through Cellular Metabolism

Using lactylome profiling, Li and colleagues provided mechanistic insights into how elevated ALDH1A3 expression promotes lactate accumulation in glioblastoma (GBM), ultimately contributing to resistance to radiotherapy and chemotherapy [127]. The study found that ALDH1A3 interacts with the glycolytic enzyme pyruvate kinase M2 (PKM2) and promotes PKM2 tetramerization, thereby enhancing its activity and increasing lactate levels in tumor cells [127]. Elevated lactate levels then induce lactylation of the DNA repair factor XRCC1 at K247, facilitating its interaction with importin α and promoting nuclear localization [127] (Figure 4, ①). This mechanism enhances glioblastoma cells’ ability to repair DNA damage caused by radiotherapy and chemotherapy, thereby contributing to greater therapeutic resistance [127].

PARP1 plays an important role in DNA damage repair. In a study of PARP1 lactylation, researchers showed that lactylation modulates its ADP-ribosylation activity [81]. Lactylation of PARP1 within its automodification domain (residues 373–524) was associated with increased ADP-ribosylation activity, and the identified lactylation sites overlap with previously reported acetylation sites that negatively regulate PARP1 activity [81]. By integrating chemical proteomic analysis with previously identified acetylation sites, the study identified seven lysine residues (K498, K505, K506, K508, K518, K521, and K524) as lactylation sites within this domain [81]. These findings suggest that PARP1 lactylation may modulate its ADP-ribosylation activity by influencing the functional effects of lysine acetylation at overlapping sites. The authors propose that this regulation may contribute to responses to DNA damage and repair. However, direct functional evidence for the role of DNA repair was not provided [81] (Figure 4, ⑦).

Notably, the lactylation’s effects on PARP1-mediated DNA damage repair are not exclusively stimulatory. Recent studies have shown that RIG-I (DDX58) undergoes PCAF-mediated lactylation [134]. Syrosingopine blocks lactate export, increasing intracellular lactate levels and promoting RIG-I lactylation [134]. This modification facilitates importin-8-dependent nuclear translocation of lactylated RIG-I [134]. Once in the nucleus, RIG-I interacts with PARP1 and inhibits its activity, thereby impairing DNA damage repair [134] (Figure 4, ⑥). This mechanism increases the sensitivity of lung adenocarcinoma cells to PARP inhibitors [134]. Thus, unlike several other lactylated DNA repair factors, RIG-I lactylation can negatively regulate DNA repair by altering RIG-I subcellular localization and its functional interaction with PARP1 [134].

Importantly, the RIG-I/PARP1 axis shows that lactylation does not always promote DNA repair. In lung adenocarcinoma models, RIG-I lactylation inhibited DNA damage repair by suppressing PARP1 activity, in contrast to the pro-repair effects reported for several other DNA repair proteins [134]. One possible explanation is that the effect of lactylation depends on the protein modified, the site of modification, and the cellular conditions under which lactylation occurs. The RIG-I study is particularly relevant here because the MCT1/4 inhibitor syrosingopine blocked lactate efflux, increased intracellular lactate, and enhanced RIG-I lactylation [134]. Thus, the effects of lactylation on DNA repair may vary between substrates and cellular settings rather than following a single direction. It remains unclear whether similar inhibitory effects occur in other DNA repair pathways or under different metabolic conditions.

In DDR, different PTMs can interact to regulate DNA repair. For example, following DNA damage, TIP60-mediated acetylation of H2AX at K5 facilitates subsequent ubiquitination of H2AX at K119, thereby promoting H2AX release from damaged chromatin and contributing to chromatin dynamics [157]. This finding illustrates that different PTMs can act sequentially and interact as part of DDR regulation rather than functioning independently. Similar crosstalk may also involve lactylation. As discussed above, PARP1 lactylation occurs at multiple lysine residues within its automodification domain. This overlaps with previously identified acetylation sites, and lactylation and acetylation exert opposing effects on PARP1 ADP-ribosylation activity [81]. These findings raise the possibility that lactylation may crosstalk with other lysine modifications through overlapping modification sites or functional coupling. However, the molecular mechanisms underlying such Kla–Kac crosstalk, as well as potential interactions between lactylation and ubiquitination in DDR proteins, remain incompletely understood and require further investigation.

Beyond tumor-intrinsic DNA damage responses, lactate/lactylation-associated signaling may also indirectly influence DNA repair by regulating the tumor immune microenvironment. Recent studies have implicated lactylation in regulating BRCA1/RAD51-associated DNA repair [57] and immune-cell functions, including macrophage M2 polarization [158] and CD8+ T-cell exhaustion [159]. However, the mechanistic connections between these processes remain incompletely defined and require further validation by direct experimental studies.

## 4. Summary and Prospects

Recent research has provided increasing evidence that Kla, a newly recognized protein post-translational modification, is an important regulator of the DDR. Earlier studies revealed that DNA damage events, including DSBs, cause dynamic changes in cellular lactylation levels. As such, Kla may provide a regulatory link between cellular metabolic status and genome stability. However, how DNA damage alters protein lactylation and how this modification is regulated within DDR remain poorly understood.

Advances in high-resolution mass spectrometry, proteomics, and lactylome profiling have enabled the identification of more lactylation sites and functional substrates. These results provide broader insights into the connections between cellular metabolism, lactylation, and DNA repair. Recent studies show that lactylation is not only associated with changes in cellular metabolic states but can also directly affect DNA damage signaling and DNA repair. Taken together, these findings suggest that lactylation may regulate different DNA repair pathways by altering the stability, interactions, and activities of repair factors. It may therefore contribute to the maintenance of genome stability.

Despite recent progress in understanding how lactylation contributes to DNA damage repair, this area of research is in its early stages, with several key questions unresolved. It remains to be determined whether distinct forms of DNA damage or endogenous and exogenous stresses induce different lactylation patterns.

It is also unclear whether lactylation contributes to damage recognition or influences the DNA repair pathway chosen. Another key question is how different lactyltransferases and delactylases work together to control lactylation in a spatially and temporally regulated manner. Further studies are needed to define the substrate specificity, regulatory mechanisms, and functional roles of these enzymes under different physiological and pathological conditions.

Technical limitations should also be considered when interpreting current evidence for protein lactylation. The specificity of anti-Kla antibodies should be carefully evaluated, particularly when lactylation is assessed mainly by immunoblotting or immunoprecipitation [71]. Although mass spectrometry has enabled the identification of numerous lactylated proteins and sites, distinguishing K_L-la from K_D-la and Kce remains challenging because of their structural similarity [71]. In addition, lysine-substitution mutants do not fully reproduce the chemical properties of native lactylated lysine residues, which may complicate the interpretation of site-specific functional studies [78]. Only a limited number of Kla reader domains have been characterized, and whether similar recognition mechanisms operate on non-histone DDR proteins remains unclear [86]. These limitations should be considered when interpreting the biological functions attributed to Kla.

Although much of the current research on Kla has focused on cancer, emerging evidence suggests that Kla is also involved in aging, neurodegenerative diseases, immune regulation, and metabolic disorders [160,161,162,163]. These studies suggest that Kla may influence cellular senescence, inflammatory responses, mitochondrial function, and metabolic homeostasis in these contexts [160,161,162,163]. However, whether and how Kla regulates DNA damage responses in non-cancer settings remains largely unclear and warrants further investigation.

Future studies should determine whether different types of DNA damage and metabolic conditions result in distinct Kla patterns and whether these changes differentially affect DNA repair. It is also important to clarify how metabolic signals regulate Kla following DNA damage and how individual Kla sites contribute to the efficiency and choice of DNA repair pathway. Defining the enzymes, substrates, and regulatory mechanisms involved in these processes may help establish Kla’s physiological significance in the DDR and clarify its potential relevance to disease.

## Figures and Tables

**Table 1:** Protein lysine lactylation in DNA damage response and repair.

Protein	Lactylation Site(s)	Writer	Eraser	Functional Effect	Disease Relevance	Ref.
MRE11	K673	CBP	SIRT1, SIRT2	Enhances DNA-binding capacity, promotes DNA end resection and homologous recombination repair	Regulates genome stability and may contribute to tumor therapeutic resistance	[55]
NBS1	K388	TIP60	HDAC3	Promotes interaction with MRE11, enhances MRN complex assembly and recruitment to DSB sites, facilitating HDR repair	Associated with chemotherapy resistance and poor clinical outcome	[54]
BLM	K24	AARS1	——	Enhances BLM stability, suppresses MIB1-mediated degradation, and promotes homologous recombination repair	Associated with anthracycline resistance in cancer cells	[122]
XLF	K288	GCN5	SIRT1	Strengthens interaction with Ku80, stabilizes Ku–XLF complex, and promotes NHEJ repair	Chemoresistance and tumor progression	[123]
XRCC1	K247	——	——	Promotes interaction with importin α and nuclear localization, enhancing DNA repair capacity	Contributes to radiotherapy and chemotherapy resistance in glioblastoma	[124]
PARP1	K498, K505, K506, K508, K518, K521, K524	——	——	Enhances ADP-ribosylation activity and may promote DNA repair	Potential involvement in DDR regulation; direct repair function requires further validation	[73]
p53	K120, K139	AARS1	——	Reduces DNA-binding ability, liquid–liquid phase separation, and transcriptional activity	Promotes tumorigenesis by suppressing p53 tumor suppressor function	[86]
RBM14	K600	——	——	Alters antiviral signaling function	May regulate antiviral immune responses during viral infection	[135]
IFI16	K90	——	——	Impairs recruitment of active DNA-PK to viral DNA and reduces cytokine production	Herpesvirus immune evasion	[135]
RIG-I	K518 K657	PCAF	——	Promotes importin-8-dependent nuclear translocation and inhibits PARP1 activity, thereby impairing DNA repair	Lung adenocarcinoma; PARP inhibitor sensitivity	[131]

Abbreviations: AARS1, alanyl-tRNA synthetase 1; CBP, CREB-binding protein; DSB, DNA double-strand break; DDR, DNA damage response; GCN5, general control of amino acid synthesis 5; HDR, homology-directed repair; HR, homologous recombination; MIB1, mindbomb E3 ubiquitin protein ligase 1; MRN, MRE11–RAD50–NBS1 complex; NHEJ, non-homologous end joining; PCAF, p300/CBP-associated factor; PARP1, poly(ADP-ribose) polymerase 1; TIP60, Tat-interactive protein 60 kDa; XLF, XRCC4-like factor. “——“ indicates that the corresponding writer or eraser has not been identified or experimentally established. The disease relevance summarizes reported or proposed associations with disease progression, therapeutic response, or treatment resistance.

## Data Availability

No new data were created or analyzed in this study. Data sharing is not applicable to this article.
